# Metabolic Amplification in Endometrial Carcinogenesis: Biological Rationale and Translational Limits of Precision Chemoprevention

**DOI:** 10.3390/biomedicines14040863

**Published:** 2026-04-09

**Authors:** Weronika Rzeska, Aneta Adamiak-Godlewska

**Affiliations:** 1 Doctoral School of Medical Sciences, Lublin Medical University, 20-093 Lublin, Poland; 2 IInd Chair and Department of Gynaecology, Lublin Medical University, 20-090 Lublin, Poland; aneta.adamiak-godlewska@umlub.pl

**Keywords:** endometrial cancer, metabolic amplification, insulin resistance, PI3K pathway, precision chemoprevention, endometrial intraepithelial neoplasia, bariatric surgery, molecular stratification

## Abstract

Background: Endometrial cancer (EC) is the most common gynecologic malignancy in developed countries and one of the few solid tumors with a steadily rising incidence, paralleling global trends in obesity and insulin resistance. Its strong epidemiologic association with systemic metabolic dysfunction positions EC as a uniquely accessible model for metabolically informed chemoprevention. Methods: This narrative review was conducted through a systematic search of PubMed/MEDLINE and Embase using the following terms: “endometrial cancer” AND (“insulin resistance” OR “metabolic syndrome” OR “PI3K” OR “chemoprevention” OR “bariatric surgery” OR “metformin” OR “cellular senescence”). Searches were limited to English-language publications; no date restriction was applied for foundational molecular studies, while clinical and translational evidence was reviewed from 2000 to 2025. Additional references were identified through manual review of reference lists of included articles. Results: We examine metabolic amplification as a conceptual framework in which hyperinsulinemia, inflammatory reinforcement, and redox-epigenetic modulation intensify proliferative signaling in biologically susceptible endometrial tissue, particularly within molecular subtypes enriched for PI3K pathway activation such as tumors lacking a specific molecular profile (NSMP). Bariatric surgery offers the strongest human evidence supporting the principle that durable metabolic correction can substantially reduce EC incidence. In contrast, pharmacologic interventions including metformin, anti-inflammatory agents, and nutraceutical compounds demonstrate variable or limited preventive efficacy, and short-term biomarker modulation cannot substitute for validated reduction in cancer risk. The endometrial intraepithelial neoplasia (EIN) model provides a uniquely accessible platform for biomarker-guided intervention. Conclusions: Integration of genomic subtype classification with metabolic profiling may enable precision prevention strategies in clearly defined high-risk populations. Effective chemoprevention will require molecular enrichment, confirmation of tissue-level target engagement, and clinically meaningful endpoints, while acknowledging the translational limits of pathway-directed approaches.

## 1. Introduction

Endometrial cancer (EC) is the most common gynecologic malignancy in developed countries and one of the few solid tumors with a steadily rising incidence [1,2,3,4]. This increase parallels global trends in obesity, insulin resistance, and metabolic syndrome, positioning EC at the intersection of systemic metabolic dysfunction and hormonally responsive tissue biology [2,3,4,5]. Epidemiologic analyses consistently demonstrate a strong association between body mass index and EC risk, with dose-dependent increases observed across prospective cohorts and meta-analyses [1,4,5]. These observations have led to the recognition of EC as one of the malignancies most tightly linked to modifiable metabolic exposure.

Despite this epidemiologic clarity, EC is biologically heterogeneous. Integrated genomic characterization has defined four principal molecular subtypes with distinct prognostic and therapeutic implications [6]. Among these, tumors lacking a specific molecular profile (NSMP)-a category operationally defined by the absence of *POLE* ultramutation, mismatch repair deficiency, and p53 abnormality-frequently harbor *PTEN* loss and related alterations in the PI3K signaling cascade [7,8,9,10]. In contrast, *POLE*-ultramutated and mismatch repair-deficient tumors are driven predominantly by genomic instability and immune-mediated mechanisms [6]. This molecular diversity suggests that metabolic influences are unlikely to act uniformly across all EC subtypes.

Systemic hyperinsulinemia, adipokine imbalance, and chronic low-grade inflammation may intensify proliferative signaling in genetically susceptible endometrial tissue [11,12,13,14,15]. Rather than functioning as primary mutagenic events, these exposures may amplify oncogenic signaling pathways already primed by *PTEN* loss or PI3K activation. The concept of metabolic amplification therefore proposes that endocrine-metabolic disturbance quantitatively enhances signaling output in hormonally responsive, pathway-activated tissue. This framework is proposed as a conceptual synthesis, distinct from general obesity-driven carcinogenesis, in that it emphasizes dependence on pre-existing molecular lesions (particularly *PTEN* loss and PI3K activation) rather than acting as an independent initiating event. Operationally, this model predicts that metabolically susceptible populations for precision chemoprevention would be identified through: (a) molecular stratification by NSMP/*PTEN* status; (b) serial assessment of insulin resistance and inflammatory biomarkers; and (c) tissue-level pharmacodynamic endpoints in EIN or presurgical window-of-opportunity trial designs.

Although this convergence provides a compelling biological rationale for prevention, translation into effective chemopreventive strategies has proven challenging. Short-term pharmacologic interventions targeting insulin signaling or inflammatory pathways have yielded heterogeneous results [16,17,18], and experiences from broader cancer prevention trials caution against overreliance on surrogate biomarker modulation. These translational limits underscore the need for molecular stratification and biologically enriched study populations.

In this review, we examine metabolic amplification as an integrative framework linking systemic dysfunction with tumor-intrinsic vulnerability. Particular emphasis is placed on PI3K pathway-enriched disease, inflammatory reinforcement, redox-epigenetic modulation, and the role of the endometrial intraepithelial neoplasia (EIN) platform in biomarker-guided prevention research [19,20].

### Review Methodology

This narrative review was conducted through a systematic search of PubMed/MEDLINE and Embase databases. Search terms included: “endometrial cancer” AND (“insulin resistance” OR “hyperinsulinemia” OR “metabolic syndrome” OR “PI3K” OR “*PTEN*” OR “mTOR” OR “chemoprevention” OR “bariatric surgery” OR “metformin” OR “inflammation” OR “adipokine” OR “epigenetic”). The search was limited to English-language publications. For foundational molecular and genomic studies, no date restriction was applied. Clinical and translational evidence was reviewed for the period 2000–2025. Additional references were identified through manual review of reference lists. Studies were selected based on predefined inclusion criteria: English-language publications; relevance to the metabolic amplification framework in endometrial carcinogenesis; clinical intervention data in EC or high-risk populations; or translational chemoprevention principles applicable to EC. Non-English publications, studies with no mechanistic or clinical relevance to EC, and case reports were excluded. Systematic reviews, meta-analyses, randomized controlled trials, and landmark mechanistic studies were prioritized where available. When conflicting evidence was identified, findings from both sides are presented with explicit discussion of methodological limitations. The initial search identified approximately 150–170 potentially relevant titles and abstracts; approximately 69 were included in the final manuscript following full-text review. Additional references were identified through manual review of reference lists of included articles. The literature search and initial selection were conducted by the first author (W.R.), with review and verification by the senior author (A.A.-G.). The narrative format and single-team screening design represent methodological limitations, which are acknowledged in the Limitations section.

## 2. Metabolic Amplification and PI3K Signaling in Endometrial Carcinogenesis

**Figure 1:**
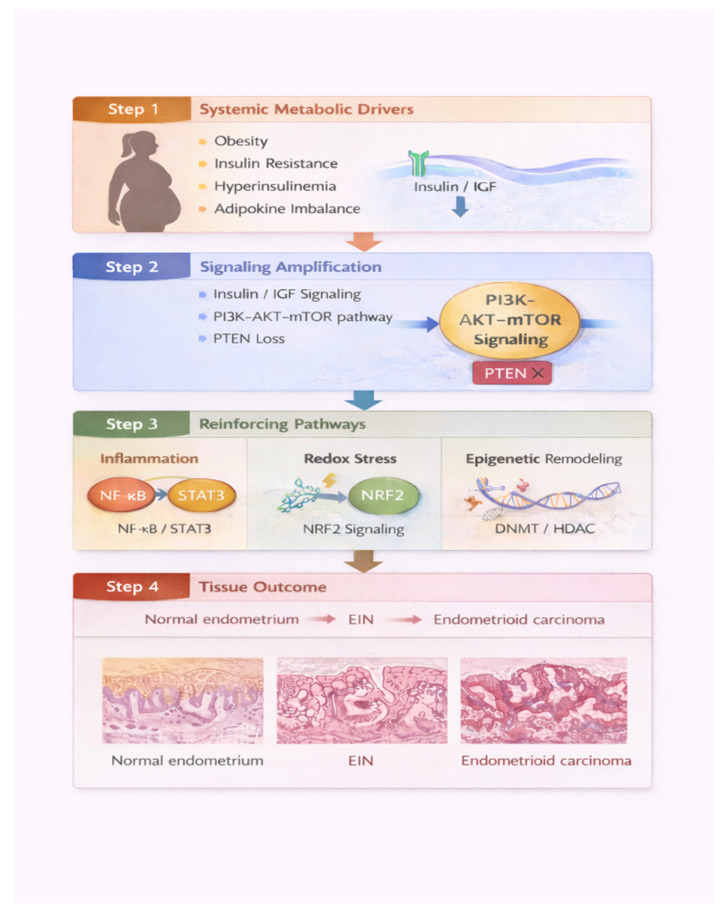
Metabolic amplification in endometrial carcinogenesis. Schematic model illustrating the proposed framework of metabolic amplification in endometrial carcinogenesis. Systemic metabolic dysfunction encompassing obesity, insulin resistance, hyperinsulinemia, and adipokine imbalance (reduced adiponectin, elevated leptin) enhances insulin/IGF-1 receptor signaling and amplifies PI3K-AKT-mTOR pathway activity. This amplification is particularly relevant in the context of pre-existing PTEN loss or PI3K pathway activation, characteristic of NSMP endometrioid tumors, where endocrine-metabolic inputs may quantitatively increase signaling flux through an already dysregulated pathway rather than acting as independent initiating events. Additional reinforcing mechanisms including inflammatory signaling (NF-κB/STAT3), oxidative stress adaptation (NRF2), and epigenetic remodeling (DNMT/HDAC) further stabilize and sustain proliferative signaling. Causal relationships between these metabolic inputs and carcinogenic progression remain incompletely established; the diagram depicts proposed amplifying interactions rather than confirmed causal drivers. These interacting processes may facilitate stepwise progression from normal endometrium through endometrial intraepithelial neoplasia (EIN) to endometrioid carcinoma, providing a biological rationale for metabolically informed, molecularly stratified chemoprevention strategies.

Metabolic dysregulation contributes to endometrial carcinogenesis primarily through amplification of proliferative signaling rather than direct mutagenesis. Hyperinsulinemia, a central feature of insulin resistance, increases circulating levels of insulin and bioavailable insulin-like growth factors, leading to enhanced activation of insulin receptor (IR) and IGF-1 receptor (IGF-1R) signaling in endometrial epithelial cells [11,12,13,14,15]. Downstream activation of the PI3K-AKT-mTOR axis promotes cellular growth, survival, and metabolic reprogramming, creating a permissive environment for neoplastic expansion (Figure 1).

Key molecular determinants contributing to metabolic amplification in endometrial cancer are summarized in Table 1.

The PI3K pathway represents one of the most frequently altered signaling cascades in endometrioid endometrial carcinoma [13,14,15]. *PTEN* loss, present in a substantial proportion of premalignant lesions and invasive tumors, reduces negative regulation of PI3K signaling and lowers the threshold for AKT activation [7,8,9,10]. Mutations in PIK3R1 and related components further destabilize regulatory control and potentiate downstream mTOR signaling [9,13,14]. In this molecular context, systemic hyperinsulinemia may act as a reinforcing stimulus, potentially increasing signaling flux through an already deregulated pathway.

Importantly, *PTEN* inactivation is frequently observed in endometrial intraepithelial neoplasia and may precede invasive transformation [7,8,20,21]. This temporal sequence is consistent with the hypothesis that metabolic amplification may operate early in carcinogenesis, intensifying proliferative signaling within genetically primed glands. Rather than serving as an initiating mutational event, insulin-mediated signaling may accelerate clonal expansion and promote progression in lesions with pre-existing pathway vulnerability.

Experimental and translational studies further support this framework. Activation of AKT and mTOR downstream effectors has been demonstrated in *PTEN*-deficient endometrial tissue, and cross-talk between insulin signaling and PI3K pathway activation enhances proliferative output and resistance to apoptosis [13,14,15,16,17,18]. AMPK-dependent modulation of mTOR signaling represents one proposed mechanism by which metabolic interventions such as metformin may counteract this amplification [16,17,18].

Presurgical window-of-opportunity trials (short-term pharmacologic interventions administered between diagnosis and planned surgery, enabling paired pre- and post-treatment tissue sampling to assess pharmacodynamic activity) have attempted to evaluate whether pharmacologic modulation of insulin signaling translates into measurable reductions in tumor proliferation. Short-term metformin exposure has been associated with variable decreases in Ki-67 expression and alterations in downstream signaling markers in some studies [16,17,18]. However, randomized data have been heterogeneous, and consistent anti-proliferative effects across molecular subtypes have not been established [18]. These findings highlight the complexity of translating systemic metabolic modulation into durable pathway suppression.

Taken together, available evidence supports a model in which metabolic amplification operates as a context-dependent enhancer of oncogenic signaling. Tumors enriched for PI3K pathway dysregulation, particularly NSMP endometrioid cancers characterized by *PTEN* loss, may be especially susceptible to endocrine reinforcement [6,15]. It is important to note, however, that NSMP is an operationally defined category characterized by the absence of *POLE* ultramutation, mismatch repair deficiency, and p53 abnormality and encompasses considerable biological heterogeneity. The association between NSMP and metabolic susceptibility reflects the enrichment of *PTEN* loss and PI3K activation within this category rather than a uniform metabolic dependency across all NSMP tumors. Subtype-specific prevention strategies targeting NSMP patients based on metabolic vulnerability should therefore be regarded as a working hypothesis requiring prospective validation rather than an established prevention paradigm. This subtype-specific vulnerability nonetheless underscores the importance of molecular stratification in future prevention-oriented studies.

## 3. Inflammatory Reinforcement and Adipokine Signaling

The role of NF-κB, STAT3, and related inflammatory pathways in oncogenesis is well established and has been extensively reviewed elsewhere. The evidence base for these mechanisms is largely experimental and derived from non-EC cancer models, and many of the associations described in this section are mechanistically plausible rather than EC-specific. The purpose of this section is therefore not to recapitulate canonical inflammatory oncology, but to contextualize these pathways within the metabolic amplification framework, specifically to examine how obesity-driven inflammatory signaling may interact with and reinforce the PI3K-enriched molecular environment that characterizes metabolically susceptible endometrial tissue.

Obesity-associated endometrial carcinogenesis may be further shaped by chronic low-grade inflammation. Expansion of visceral adipose tissue promotes secretion of pro-inflammatory cytokines, including interleukin-6 (IL-6), tumor necrosis factor-α (TNF-α), and other mediators that activate transcriptional programs through NF-κB and STAT3 signaling pathways [22,23,24]. These inflammatory cascades intersect with insulin-mediated PI3K-AKT activation and may reinforce proliferative and anti-apoptotic signaling within metabolically susceptible endometrial epithelium.

NF-κB signaling functions as a central regulator of inflammation-associated oncogenesis and may promote transcription of genes involved in cell survival, angiogenesis, and immune modulation [22,23]. Persistent activation of this pathway in obesity may sustain a microenvironment conducive to tumor promotion. Similarly, IL-6-mediated activation of the JAK/STAT axis has been implicated in enhanced proliferation, epithelial–mesenchymal transition, and acquisition of stem-like characteristics in endometrial carcinoma models [25,26]. The convergence of inflammatory and metabolic signaling pathways may contribute to increased oncogenic signaling in a manner that may be particularly relevant in PI3K-activated disease.

Cyclooxygenase-2 (COX-2) expression and downstream prostaglandin signaling represent additional inflammatory mediators implicated in endometrial tumor biology [25,26,27,28,29,30]. Elevated COX-2 expression has been detected in endometrial carcinomas and is associated with enhanced angiogenesis and local immune modulation in observational studies [28]. Tumor-associated macrophages (TAMs), enriched in obesity-associated microenvironments, may contribute to cytokine production and correlate with adverse clinicopathologic features in observational data [26,27]. The presence of TAMs may amplify proliferative signaling and facilitate immune evasion, reinforcing the concept of inflammatory amplification in metabolically dysregulated tissue.

Adipokine imbalance provides an additional mechanistic bridge between systemic metabolic dysfunction and local tumor biology. Reduced circulating adiponectin levels and elevated leptin concentrations have been associated with increased EC risk in observational studies [31,32,33]. Adiponectin exerts insulin-sensitizing and anti-inflammatory effects, whereas leptin can activate PI3K, MAPK, and JAK/STAT pathways, potentially promoting proliferation and angiogenesis. This imbalance may further intensify signaling in tissue already characterized by *PTEN* loss or PI3K pathway activation.

Given these biological links, anti-inflammatory agents have been explored as potential chemopreventive interventions. Observational data suggest possible protective associations between nonsteroidal anti-inflammatory drugs (NSAIDs), including aspirin, and EC risk or prognosis, although findings remain heterogeneous and largely hypothesis-generating [29,30]. Broader cancer prevention literature has demonstrated that COX inhibition may reduce incidence of certain malignancies; however, extrapolation to EC requires caution and prospective validation [34].

Collectively, inflammatory reinforcement may operate in parallel with endocrine amplification, creating a signaling environment in which proliferative pathways are persistently stimulated. The magnitude and clinical relevance of this interaction likely vary across molecular subtypes. In tumors driven primarily by genomic instability, metabolic-inflammatory reinforcement may play a less central role, whereas in hormonally responsive, PI3K-enriched disease, the convergence of these pathways may substantially influence progression dynamics [6,7,8,9,10].

## 4. Redox-Epigenetic Modulation and Nutraceutical Interventions

Beyond endocrine and inflammatory reinforcement, metabolic dysfunction may influence endometrial carcinogenesis through redox imbalance and epigenetic remodeling. Obesity is associated with increased oxidative stress, mitochondrial dysfunction, and altered cellular redox homeostasis. These disturbances can modulate signaling pathways governing proliferation, apoptosis, and DNA repair. Activation of NRF2-dependent antioxidant transcriptional programs represents a central adaptive response to oxidative stress and may influence tumor behavior within metabolically dysregulated environments [35,36,37]. Persistent oxidative signaling may further interact with PI3K-AKT and inflammatory cascades, reinforcing proliferative output.

Metabolic-epigenetic coupling provides an additional mechanistic bridge between systemic metabolic status and gene expression regulation. Availability of metabolic intermediates such as acetyl-CoA, S-adenosylmethionine, and NAD^+^ can influence histone acetylation, DNA methylation, and chromatin remodeling. Epigenome-wide association studies have demonstrated obesity-related DNA methylation changes in multiple tissues, supporting the plausibility of metabolically driven epigenetic remodeling [38,39]. In endometrial tissue, such alterations may modify transcriptional programs involved in proliferation, differentiation, and inflammatory signaling, although direct causal links to malignant progression remain incompletely defined.

These biological considerations have generated preliminary interest in nutraceutical compounds with proposed redox and epigenetic activity. Sulforaphane, a naturally occurring isothiocyanate derived from cruciferous vegetables, functions in part as a histone deacetylase inhibitor and has demonstrated anti-proliferative activity in preclinical cancer models [40,41,42,43]. Experimental studies in endometrial cancer systems suggest potential modulation of cell-cycle regulators and apoptotic pathways [41]. Similarly, epigallocatechin gallate (EGCG), a polyphenol found in green tea, has been shown to inhibit DNA methyltransferase activity and influence oncogenic signaling networks [44,45,46]. Curcumin, another bioactive compound with anti-inflammatory and epigenetic properties, has demonstrated mechanistic activity in vitro; however, its clinical translation is limited by poor bioavailability and pharmacokinetic constraints [35,36,37]. It should be noted that the majority of preclinical data for these compounds derives from cancer models other than endometrial cancer; findings from in vitro or animal studies in colorectal, breast, or prostate cancer cannot be directly extrapolated to EC without dedicated experimental and clinical validation in endometrial-specific systems.

Despite promising mechanistic data, clinical evidence supporting nutraceutical interventions as effective chemopreventive agents in EC remains limited and largely indirect. Surrogate biomarker modulation, such as changes in Ki-67, inflammatory mediators, or epigenetic marks, does not necessarily translate into meaningful reductions in cancer incidence. Experiences from large randomized prevention trials in other malignancies underscore the limitations of antioxidant supplementation strategies, which failed to reduce cancer risk and, in some instances, increased incidence. These findings caution against extrapolating short-term molecular effects to long-term preventive benefit without robust clinical validation [47,48].

Redox-epigenetic modulation therefore represents a biologically plausible but as yet unproven axis of intervention. Future studies must incorporate molecular stratification, rigorous pharmacodynamic assessment, and clinically relevant endpoints to determine whether modulation of oxidative or epigenetic pathways can meaningfully alter the trajectory of metabolically amplified endometrial carcinogenesis.

## 5. Clinical Evidence: Established Strategies, Pharmacologic Agents Under Investigation, and Exploratory Approaches

### 5.1. Established and Clinically Validated Strategies

Among available strategies, bariatric surgery represents a surgical treatment for obesity that achieves global and durable metabolic normalization, and provides the most compelling human evidence that sustained metabolic correction can reduce endometrial cancer risk. Large population-based cohorts and systematic reviews consistently demonstrate substantial reductions in EC incidence following significant and durable weight loss [49,50,51]. These findings extend across diverse populations and support the principle that long-term normalization of insulin levels, adipokine balance, and inflammatory signaling may meaningfully alter carcinogenic trajectories. The magnitude of risk reduction observed after bariatric surgery exceeds that reported for most pharmacologic or nutraceutical interventions, underscoring the importance of durable systemic metabolic modification.

### 5.2. Pharmacologic Agents Under Investigation

In contrast, pharmacologic metabolic modulation has yielded more heterogeneous results. Metformin is mechanistically plausible as a metabolic intervention—it activates AMPK and suppresses mTOR signaling—but its preventive efficacy in EC has not been established. Observational studies initially suggested that metformin use among patients with type 2 diabetes may be associated with reduced overall cancer incidence and improved oncologic outcomes [52,53,54]. However, observational designs are susceptible to confounding and immortal time bias. Randomized data specifically addressing EC prevention remain limited, and the preventive efficacy of metformin in metabolically stratified but non-diabetic populations has not been conclusively established [16,17,18]. Short-term presurgical trials demonstrate variable reductions in proliferative markers such as Ki-67 and modulation of AMPK-mTOR signaling, yet durable clinical benefit remains unproven.

Nonsteroidal anti-inflammatory drugs (NSAIDs), including aspirin, have been explored based on observational data suggesting possible protective associations with EC risk or prognosis. However, these findings remain heterogeneous and largely hypothesis-generating; no EC-specific randomized trial has evaluated NSAIDs as chemopreventive agents, and extrapolation from colorectal cancer data requires caution [29,30]. It should be noted that no pharmacologic or nutraceutical agent currently meets established criteria for evidence-based endometrial cancer chemoprevention outside of hormonal strategies.

Emerging glucose-lowering agents have also been evaluated with respect to malignancy risk. Large cardiovascular outcome trials and meta-analyses examining GLP-1 receptor agonists and SGLT2 inhibitors have not demonstrated consistent increases in overall cancer incidence [53,54]. While reassuring from a safety perspective, these studies were not designed to assess cancer prevention in enriched EC populations. Dedicated trials incorporating molecular stratification and tissue-level endpoints would be required to evaluate preventive efficacy in endometrial carcinogenesis.

### 5.3. Established Hormonal Strategies and Exploratory Approaches

Hormonal prevention strategies remain among the most robustly supported interventions in EC. Comprehensive collaborative meta-analyses confirm a sustained protective association between combined oral contraceptive use and reduced endometrial cancer risk, with benefit persisting for years after discontinuation [55,56]. These data highlight the central role of endocrine modulation in hormonally responsive tissue and provide a benchmark against which metabolically oriented interventions may be compared.

For women with endometrial intraepithelial neoplasia, progestin-based therapies including oral progestogens and the levonorgestrel-releasing intrauterine system (LNG-IUS) demonstrate high rates of histologic regression and represent established fertility-sparing management strategies [19,20,21,57,58,59,60]. While effective in treating premalignant lesions, these interventions primarily target local progesterone responsiveness rather than systemic metabolic drivers. Nonetheless, the EIN platform offers a valuable setting for biomarker-guided translational research and assessment of tissue-level pharmacodynamic effects.

Lessons from large-scale chemoprevention programs in other malignancies caution against overinterpretation of surrogate biomarker changes. Randomized trials of antioxidant supplementation, including beta-carotene, vitamin A, vitamin E, and selenium, failed to reduce cancer incidence and, in some cases, increased risk. Broader chemoprevention frameworks emphasize the importance of validated intermediate endpoints, molecular enrichment, and rigorous trial design. These principles are directly applicable to EC prevention research, where pathway-directed interventions must demonstrate durable clinical benefit rather than short-term modulation of signaling markers.

It should be noted that no pharmacologic or nutraceutical agent currently meets established criteria for evidence-based endometrial cancer chemoprevention outside of hormonal strategies. All pharmacologic agents discussed in Section 5.2 and exploratory approaches in Section 5.3 represent investigational strategies requiring prospective validation in molecularly enriched, EC-specific prevention trials before clinical recommendation can be considered.

Collectively, current clinical evidence supports the concept that durable systemic metabolic normalization can reduce EC risk, while pharmacologic and nutraceutical approaches require more robust validation. Precision chemoprevention strategies must therefore integrate molecular subtype classification, metabolic profiling, and rigorous translational methodology to determine which patient populations are most likely to benefit.

## 6. Discussion

Endometrial cancer occupies a distinctive position among solid tumors in that its incidence closely mirrors global trends in obesity and metabolic dysfunction [1,2,3,4,5]. Few malignancies demonstrate such a strong and consistent epidemiologic association with modifiable endocrine-metabolic exposure. Yet despite this clarity, translation of metabolic risk into validated chemopreventive strategies has been incremental and complex.

The metabolic amplification model provides a conceptual framework for integrating systemic metabolic disturbance with tumor-intrinsic signaling vulnerability. In tumors enriched for PI3K pathway activation, particularly NSMP endometrioid cancers characterized by *PTEN* loss, hyperinsulinemia may quantitatively enhance signaling through the PI3K-AKT-mTOR axis rather than initiate oncogenesis independently [6,7,8,9,10,11,12,13,14,15]. This distinction may be important. Amplification implies context dependence: endocrine reinforcement is most likely to influence progression in tissue already primed by pathway dysregulation.

Durable metabolic correction, exemplified by bariatric surgery, offers the strongest human evidence supporting this framework [49,50,51]. The magnitude and consistency of EC risk reduction observed after substantial weight loss contrast with the more modest and heterogeneous results reported for pharmacologic metabolic modulation. Metformin illustrates this translational complexity. While mechanistic studies demonstrate AMPK activation and downstream mTOR suppression [16,17,18], and short-term presurgical trials suggest variable anti-proliferative effects [16,17,18], consistent long-term preventive benefit has not been established.

Inflammatory reinforcement further complicates this landscape. Obesity-associated cytokine signaling, NF-κB activation, and COX-2-mediated pathways intersect with insulin-driven PI3K signaling and may stabilize proliferative output in susceptible tissue [23,24,25,26,27,28,29,61]. However, inflammatory signatures differ across molecular subtypes. In *POLE*-ultramutated and mismatch repair-deficient tumors, immune activation is largely mutation-driven rather than metabolically mediated [6]. Thus, the contribution of inflammatory amplification likely varies according to genomic context.

Redox imbalance and epigenetic remodeling introduce additional layers of biological complexity. Although obesity-associated DNA methylation changes and metabolic-epigenetic coupling are mechanistically plausible [38,39], direct evidence linking these alterations to stepwise progression from EIN to invasive carcinoma remains limited. Nutraceutical interventions targeting redox or epigenetic pathways demonstrate intriguing preclinical activity [41,45,47,48,62,63,64,65,66], yet experiences from large antioxidant trials caution against extrapolating short-term molecular effects to durable cancer prevention.

The EIN platform represents a uniquely accessible model for translational research in EC prevention. Histologic reproducibility, molecular characterization, and measurable progression risk have been well described [19,20,21,67,68,69,70]. Progestin-based regression of EIN demonstrates that hormonally responsive lesions can be modulated therapeutically [19,20,21,69], providing proof of principle that premalignant endometrial tissue remains biologically plastic. Future prevention strategies could leverage this platform for biomarker-guided trials incorporating metabolic stratification and tissue-level pharmacodynamic assessment.

Experience from broader chemoprevention efforts reinforces the importance of rigorous trial design. Frameworks emphasizing validated intermediate biomarkers, enrichment of high-risk populations, and demonstration of clinically meaningful endpoints have been articulated extensively. These principles are directly applicable to EC, where prevention strategies must move beyond pathway plausibility toward demonstrable incidence reduction in biologically defined cohorts.

Importantly, hormonal prevention strategies remain among the most robustly supported interventions in EC. Large collaborative analyses confirm sustained protective effects of combined oral contraceptives [55,56], underscoring the potency of endocrine modulation in hormonally responsive tissue. In comparison, metabolic pharmacotherapies including GLP-1 receptor agonists and SGLT2 inhibitors act through distinct but complementary mechanisms: GLP-1 receptor agonists reduce body weight and improve insulin sensitivity through incretin-mediated pathways, while SGLT2 inhibitors lower glucose levels, reduce adiposity, and exert anti-inflammatory effects. Large cardiovascular outcome trials and meta-analyses have not demonstrated increases in overall cancer incidence with either drug class [53,54], providing reassurance regarding oncologic safety. However, these trials were not designed to evaluate cancer prevention in molecularly enriched endometrial cancer populations. The potential for indirect risk reduction through sustained metabolic normalization is biologically plausible but requires prospective evaluation in dedicated EC prevention trials incorporating molecular stratification and tissue-level endpoints.

A critical unresolved question concerns the causal status of metabolic dysfunction in endometrial carcinogenesis. Three interpretations must be distinguished. First, metabolic dysfunction may represent a genuine causal driver capable of initiating oncogenic signaling de novo. Second, it may function as a context-dependent modifier or amplifier that accelerates progression in tissue already primed by molecular lesions such as *PTEN* loss or PI3K activation—the central thesis of the metabolic amplification framework. Third, observed associations may partly reflect reverse causation or correlated epiphenomena, where metabolic abnormalities accompany but do not causally drive carcinogenesis. Whether metabolic dysfunction represents a causal driver, a context-dependent modifier, or a correlated epiphenomenon remains unresolved. Current evidence—predominantly observational, mechanistic, and derived from heterogeneous populations—cannot definitively distinguish between these possibilities. Mendelian randomization studies and molecularly stratified prospective prevention trials represent the most appropriate methodological frameworks to address this fundamental question. Until such evidence is available, the metabolic amplification model should be regarded as a compelling and operationally useful hypothesis rather than an established mechanistic principle. Ultimately, endometrial carcinogenesis is multifactorial and subtype-dependent. Metabolic dysfunction represents a major modifiable exposure but interacts with genomic architecture, inflammatory context, and hormonal signaling. Precision chemoprevention will require integration of molecular subtype classification, metabolic profiling, durable systemic correction, and carefully validated endpoints. The conceptual framework linking metabolic amplification with stepwise progression in endometrial carcinogenesis is illustrated in Figure 1. The principal translational challenges that must be addressed in future prevention studies are summarized in Table 2.

Without such integration, pathway-directed interventions risk reproducing the translational limitations observed in prior prevention efforts.

### Limitations

Several limitations of this review warrant acknowledgement. First, the narrative methodology introduces potential selection bias; studies supporting the metabolic amplification model may be disproportionately represented. Second, evidence for the metabolic amplification framework remains largely mechanistic and preclinical; direct human causal evidence linking PI3K pathway activation to metabolic inputs in prospective trials is limited. Third, NSMP is operationally defined by exclusion from three positive molecular subtypes and represents a heterogeneous category rather than a biologically uniform entity, limiting the specificity of subtype-directed prevention conclusions. Fourth, the majority of pharmacologic intervention studies reviewed here employed short-term surrogate endpoints (Ki-67 expression, phosphorylation markers) rather than validated cancer incidence outcomes; the clinical translatability of these markers remains unconfirmed. Fifth, publication bias may overrepresent positive mechanistic findings in the chemoprevention literature. These limitations are directly relevant to the translational requirements outlined in Table 2 and underscore the need for prospective, molecularly enriched prevention trials.

## 7. Conclusions

Endometrial cancer remains one of the malignancies most strongly shaped by systemic metabolic dysfunction. The concept of metabolic amplification integrates epidemiologic observation with molecular vulnerability, linking hyperinsulinemia, inflammatory reinforcement, and redox–epigenetic modulation to intensified proliferative signaling in biologically susceptible endometrial tissue [6,7,8,9,10,11,12,13,14,15]. Within PI3K pathway-activated, NSMP-enriched tumors, systemic endocrine disturbance may quantitatively enhance oncogenic signaling rather than initiate transformation de novo.

Clinical evidence indicates that durable systemic metabolic correction can meaningfully alter cancer risk trajectories. Bariatric surgery provides the strongest human proof-of-principle, demonstrating substantial and sustained reductions in endometrial cancer incidence following long-term weight loss [49,50,51]. In contrast, short-term pharmacologic interventions targeting insulin signaling, inflammatory pathways, or epigenetic modulation have not yet demonstrated consistent preventive efficacy across unselected populations [16,17,18,41,45,65,66]. These findings highlight the translational limits of pathway-directed chemoprevention when not embedded within sustained metabolic normalization.

Hormonal prevention strategies, particularly combined oral contraceptives, remain among the most robustly validated interventions for reducing EC risk [55,56], emphasizing the continued importance of endocrine modulation in hormonally responsive tissue. Emerging data regarding glucose-lowering agents including GLP-1 receptor agonists and SGLT2 inhibitors provide reassurance regarding overall malignancy risk but require prospective evaluation within molecularly enriched endometrial cancer cohorts [53,54].

Future progress in precision chemoprevention will depend on integration of genomic subtype classification with metabolic profiling, rigorous validation within premalignant platforms such as endometrial intraepithelial neoplasia, and demonstration of tissue-level target engagement prior to invasive transformation [19,20,21,22]. Established chemoprevention frameworks underscore the necessity of validated intermediate biomarkers, enrichment of biologically defined high-risk populations, and confirmation of clinically meaningful endpoints before widespread implementation.

Ultimately, meaningful reduction in endometrial cancer incidence is unlikely to arise from broad, non-stratified pathway modulation alone. Instead, prevention strategies must align mechanistic rationale with molecular context, durable systemic correction, and carefully designed translational studies capable of demonstrating long-term clinical benefit.

## Figures and Tables

**Table 1:** Molecular determinants underlying metabolic amplification in endometrial cancer.

Pathway/Axis	Functional Contribution	Evidence Base	Molecular Subtype Relevance	Preventive Consideration	Main Limitation	Strength of Evidence
Insulin/IGF-PI3K-AKT-mTOR	Sustains proliferative and anabolic signaling under hyperinsulinemic conditions	Epidemiologic + genomic + translational	Enriched in NSMP	Insulin-lowering or AMPK-directed strategies	Variable metabolic dependency	High
*PTEN* loss	Removes inhibitory control of PI3K signaling	Genomic (common in endometrioid EC)	High (NSMP-associated)	Marker of signaling susceptibility	Absent in non-endometrioid subsets	High
AMPK axis	Energetic counter-regulation of mTOR activity	Mechanistic + presurgical data	Context-dependent	Metabolic modulation (e.g., metformin)	Inconsistent biomarker response	Moderate
NF-κB/STAT3	Couples inflammatory signaling with survival pathways	Experimental + tissue studies	Likely obesity-associated	Anti-inflammatory targeting (hypothesis-driven)	No EC-specific RCT evidence	Low
NRF2-mediated redox control	Regulates oxidative stress adaptation	Preclinical	Uncertain	Early-stage theoretical relevance	Stage-dependent effects	Preclinical
Epigenetic regulators (DNMT/HDAC)	Influence transcriptional stability under metabolic stress	Experimental	Unclear	Conceptual early intervention	Limited human validation	Preclinical

**Table 2:** Translational requirements for precision chemoprevention in endometrial cancer.

Domain	Core Issue	Implication for Prevention Studies	Current Evidence Gap
Molecular diversity	Distinct genomic subtypes with divergent drivers	Subtype-based enrollment (e.g., NSMP, *PTEN* loss)	No EC subtype-specific prevention trial completed; NSMP enrichment remains a working hypothesis [6,7]
Metabolic heterogeneity	Dynamic insulin resistance and inflammatory burden	Serial metabolic assessment	No validated composite metabolic index for EC risk stratification exists; HOMA-IR and adipokine panels used in research only [11,12]
Surrogate reliance	Biomarker change may not reflect incidence reduction	Incorporation of validated clinical endpoints	All available pharmacologic intervention studies use surrogate endpoints (Ki-67, pAKT); no agent has reduced EC incidence in a randomized trial [16,17,18]
Tissue pharmacodynamics	Uncertain endometrial target engagement	EIN-based tissue monitoring	EIN platform available but underutilized for metabolically stratified prevention trials; biomarker-endpoint linkage unvalidated [19,20]
Effect magnitude	Modest signaling shifts may be insufficient	Emphasis on durable metabolic correction	Only bariatric surgery achieves durable correction with demonstrated EC risk reduction; pharmacologic equivalents not established [49,50,51]
Long-term safety	Preventive exposure requires chronic administration	Structured safety evaluation	Long-term safety data for preventive use of metabolic agents in non-diabetic EC-risk populations largely absent

## Data Availability

No new data were created or analyzed in this study.

## Abbreviations

The following abbreviations are used in this manuscript: AKT—Protein kinase B (PKB); AMPK—AMP-activated protein kinase COX-2—Cyclooxygenase-2; DNMT—DNA methyltransferase; EC—Endometrial cancer; EGCG—Epigallocatechin gallate; EIN—Endometrial intraepithelial neoplasia; GLP-1 RA—Glucagon-like peptide-1 receptor agonist; HDAC—Histone deacetylase; IGF—Insulin-like growth factor; IGF-1R—Insulin-like growth factor 1 receptor; IL-6—Interleukin-6; IR—Insulin receptor; JAK—Janus kinase; LNG-IUS—Levonorgestrel-releasing intrauterine system; mTOR—Mechanistic target of rapamycin; NF-κB—Nuclear factor kappa B; NRF2—Nuclear factor erythroid 2-related factor 2; NSMP—No specific molecular profile; PI3K—Phosphoinositide 3-kinase; PIK3R1—Phosphoinositide 3-kinase regulatory subunit 1; *PTEN*—Phosphatase and tensin homolog; RCT—Randomized controlled trial; SGLT-2i—Sodium-glucose cotransporter-2 inhibitor; STAT3—Signal transducer and activator of transcription 3; TAM—Tumor-associated macrophage; TNF-α—Tumor necrosis factor alpha.
